# Omega-3 fatty acid ameliorates bisphenol F-induced testicular toxicity by modulating Nrf2/NFkB pathway and apoptotic signaling

**DOI:** 10.3389/fendo.2023.1256154

**Published:** 2023-09-20

**Authors:** Adeyemi Fatai Odetayo, Wale Johnson Adeyemi, Luqman Aribidesi Olayaki

**Affiliations:** ^1^ Physiology Department, University of Ilorin, Ilorin, Nigeria; ^2^ Physiology Department, Federal University of Health Sciences, Ila Orangun, Osun State, Nigeria; ^3^ Physiology Department, Adeleke University, Ede, Nigeria

**Keywords:** omega-3 fatty acid, bisphenol F, bisphenol analogs, endocrine disruptors, testicular functions, apoptosis

## Abstract

**Introduction:**

Bisphenol F (BPF) has been shown to disrupt testicular functions via perturbation of testicular redox balance, while omega-3 fatty acid (O3FA) has been established to exert antioxidant and anti-inflammatory activities. Therefore, this study focused on the role and associated molecular mechanism of O3FA in BPF-induced testicular dysfunction in male Wistar rats.

**Methods:**

Twenty-four (24) rats were randomly grouped after two weeks of acclimatization into four (4) groups (n=6/group); the vehicle-treated control group, BPF treated group received 30 mg/kg of BPF, and the intervention groups received 30 mg/kg BPF + 100 mg/kg O3FA (BPF+O3FA-L) and 30 mg/kg BPF + 300 mg/kg of O3FA (BPF+O3FA-H). All treatment lasted for 28 days.

**Results:**

Low and high doses of O3FA ameliorated BPF-impaired sperm quality, and induced hormonal imbalance, accompanied by a distortion in testicular histology and elevated testicular injury markers. Furthermore, co-administration of BPF with both doses of O3FA blunted BPF-induced redox imbalance, inflammatory response, and apoptosis.

**Discussions:**

In conclusion, our present findings show that O3FA improves testicular functions in BPF-treated rats by improving sperm quality and reproductive hormones via the maintenance of testicular redox balance.

## 1 Introduction

Plastics and cans are used in almost every facet of daily life. They are utilized in transportation, telecommunications, clothes, footwear, and, most importantly, as packaging materials for various foods, beverages, and other commodities. Numerous researches have been conducted on various elements of plastics and cans, particularly their environmental effects and risks to natural environments, wildlife, and, most significantly, human health. One of the major raw materials in the production of plastics and cans is bisphenol A (BPA) (1).

BPA is ubiquitous in the environment, resulting in high rate of human exposure to this chemical. The concern of widespread exposure and established adverse effects on human health has led to strict restrictions on the production and usage of BPA in Canada, France, and the European Union in 2008, 2010, and 2011 respectively (2). This consequently led to the introduction of alternative substitutes for BPA. As the focus has switched to producing “BPA-free” products, bisphenol F (BPF) has become the major replacement for BPA. It is now widely used to produce everyday consumer products such as plastics, cans, thermal papers, and inner linings of food containers, infant bottles, and toys. Unfortunately, BPF, which is expected to be a safer alternative to BPA, displays a similar gonadotoxic effect to BPA. BPF is gradually becoming a ubiquitous chemical, and investigations have revealed that BPF may harm the reproductive system (3–5). BPF exposure has been implicated in the increased production of free radicals (oxidative stress) and pro-inflammatory cytokines (4, 6, 7), which is a major cause of testicular toxicity.

Nuclear Factor Erythroid Related Factor 2 (Nrf2) and Nuclear Factor-Kappa B (NFκB) are key regulators of the body’s response to oxidative stress and inflammatory response (8). During excessive and continuous exposure to external stresses, the body produces excess free radicals and reactive oxygen species (ROS), leading to the downregulation of endogenous antioxidants, enzymes, and proteins, thereby damaging the body’s cellular components such as proteins, DNA, and lipids (9). Nrf2 is a major endogenous antioxidant controlling various aspects of cellular homeostasis in response to oxidative stress (10). The decline in Nrf2 due to external stressors can upregulate NFκB expression, leading to an inflammatory response. Also, the increase in NFκB expression can also lead to a further decrease in Nrf2. Hence, Nrf2 and NFκB are important players in the crosstalk between oxidative stress and inflammation (11). The excessive decrease in the endogenous antioxidant system and increased inflammatory response can possibly trigger an apoptotic response (12). On the other hand, supplementation of exogenous antioxidants can target oxidative stress by inhibiting the production of free radicals and ROS and bolstering the endogenous antioxidant capacity.

Omega-3 fatty acid (O3FA) is a polyunsaturated fatty acid (PUFA) and an antioxidant with favorable effects against various diseases such as cardiovascular disorder (13) and reproductive dysfunction (14). O3FA can protect organs such as the testis via its antioxidant (15), anti-inflammatory, and antiapoptotic (14) properties. These data suggest that O3FA could be a promising cytoprotective agent against extrinsic toxic stimuli. Despite these established protective functions of O3FA, no study has investigated the effectiveness of O3FA on testicular dysfunction in BPF-induced reproductive toxicity. Hence, this study was designed to investigate the ameliorative effect of O3FA on BPF-induced gonadotoxicity.

## 2 Methods

### 2.1 Chemical

O3FA was purchased from Gujarat Liqui Pharmacaps Pvt. Ltd. Vadodara, Gujarat, India, and each O3FA capsule contains eicosapentaenoic acid (EPA) and docosahexaenoic acid (DHA) in the ratio of 3:2. BPF was purchased from Sigma-Aldrich, St. Louis, MO, USA, CAS: 620-92-8. All other chemicals except otherwise stated were purchased from Sigma Aldrich.

### 2.2 Animals

Twenty-four (24) male Wistar rats of age 10 ± 2 weeks with comparable weights (160-180 g) were obtained from the University of Ilorin. The animals were randomly separated into clean wooden cages under natural conditions and were allowed unlimited free access to feed and water ad’libitum. The designed experimental protocol was approved by the University of Ilorin Review and Ethical Committee, and in accordance with the “National Institute of Health guidelines using the guide for the care and handling of laboratory animals (NIH Publication No. 80–23; amended 1978)”. The experimental protocol was under the National Research Council’s guidelines for the Care and Use of Laboratory Animals, and ARRIVE guidelines for reporting experimental findings were followed. Animals were randomly grouped after two weeks of acclimatization into four (4) groups (n=6/group); the vehicle-treated control group, BPF treated group received 30 mg/kg of BPF, and the intervention groups received 30 mg/kg BPF + 100 mg/kg O3FA (BPF+O3FA-L) and 30 mg/kg BPF + 300 mg/kg of O3FA (BPF+O3FA-H).

### 2.3 Sample collection

The dose of BPF was calculated and dissolved in corn oil, and 0.5 ml of the solution containing the appropriate calculated dose was administered for each animal. The 28 days administrations were carried out using an oro-pharyngeal cannula via the oral route to mimic the main route of human exposure. Overnight fasted animals were sacrificed 24 hours after the last dose of BPF and O3FA with ketamine (40 mg/kg) and xylazine (4 mg/kg) i.p (16). Blood samples were collected via cardiac puncture while the left and right testes and left epididymides were harvested. The blood samples were centrifuged at 3000 rpm to obtain the serum for hormonal analysis, while the left testes were homogenized in cold Phosphate Buffer for biochemical assays. The right testes were used for histological examination, while the left epididymides were harvested for sperm analysis.

### 2.4 Epididymal sperm parameters

Each caudal epididymis was carefully cut into small pieces in a clean petri dish and sperm count, motility, and abnormal sperm morphology were determined as previously described (14, 17).

### 2.5 Reproductive hormones

Serum luteinizing hormone (LH) (Catalogue no.: B-1-121032301), follicle-stimulating hormone (FSH) (Catalogue no.: B-1-121040801), testosterone (B-1- 121071602), and estradiol (B-1-122042001) were quantified using an ELISA method following the manufacturer’s guidelines (Bio-Inteco, UK).

### 2.6 Histology

Testicular histopathological analysis was performed based on documented methods (14, 18). The testis was fixed in bouin solution, dehydrated with ethanol series, and cleared with toluene. It was then embedded at room temperature of 37°C and blocked in paraffin wax incubated overnight in a 60°C incubator. Afterward, hematoxylin and eosin (H&E) stain was applied to the testes’ 5 µm thick paraffin sections.

Testicular histoarchitecture was determined as established by Cosentino et al. (19) scoring system as follows:

“4: Irregular and distorted seminiferous tubules engorged by coagulative necrosis in the germ cells.

3: Disordered and sloughed germ cells with shrunken and pyknotic nuclei and impaired borders of the seminiferous tubules.

2: Loss of cohesion in germ cells, closely packed seminiferous tubules.

1: Normal testicular tissue with an orderly arrangement of germ cells”.

The Mean testicular biopsy score (MTBS), which is an index of spermatogenesis, was determined at 400 X microscopic field as earlier established by Johnsen (20) scoring system as follows:

“10: Complete spermatogenesis with many spermatozoa.

9: Many spermatozoa present but disorganized germinal epithelium.

8: Only a few spermatozoa (<5–10) are present.

7: No spermatozoa but many spermatids present.

6: No spermatozoa and only a few spermatids (<5–10) present.

5: No spermatozoa or spermatids but several or many spermatocytes present.

4: Only a few spermatocytes (<5) and no spermatids or spermatozoa present.

3: Spermatogonia are the only germ cells present.

2: No germ cells, but Sertoli cells are present.

1: No cells (either germ cell or Sertoli cell) in the tubular section”

Mean seminiferous tubular and luminal diameter and epithelial height were estimated as reported earlier (14, 21, 22). “Mean Seminiferous Tubular Diameter (MSTD) of each testis was determined by measuring 20 separate roundest seminiferous tubules with a light microscope-adaptable micrometer. The mean of the values obtained was regarded as the MSTD of the testis.

### 2.7 Testicular injury markers

Gamma-glutamyl transferase (GGT) activities were estimated according to the manufacturer’s instructions (Agape Diagnostics Ltd., CAT: 31070095), while Lactate dehydrogenase activities were also determined following the manufacturer’s instructions (Agape Diagnostics Ltd., CAT: 31060230) using a spectrophotometer. The testicular lactate concentration was also estimated according to the manufacturer’s guidelines (EnzyChrom, ELAC-100).

### 2.8 Steroidogenic enzymes

Testicular 3 beta-hydroxysteroid (3β-HSD) and 17 beta-hydroxysteroid (17 β-HSD) enzymatic activities were determined as previously documented (23) and (24) respectively.

### 2.9 Inflammatory markers

Standard ELISA kits were used to assay the concentrations of interleukin-6 (IL-6) (Solarbio, China, CAT: SEKH-0013) and tumour necrosis factor-α (TNF- α) (Solarbio, China, CAT: SEKH-0047), NFkB (Elabscience Biotechnology Inc., USA, CAT: E-EL-R0673) were determined using ELISA kits. Testicular Myeloperoxidase (MPO) and nitric oxide were determined based on established methods (25) and (26), respectively.

### 2.10 Markers of oxidative stress

Testicular malondialdehyde (MDA) (27) levels were assayed as previously reported. In addition, testicular glutathione (GSH), glutathione peroxidase (GPx), Glutathione-S-transferase (GST), superoxide dismutase (SOD), and catalase (CAT) (10, 14, 28) activities were assayed by colorimetric methods as previously reported. In addition, testicular Nrf2 was determined using an ELISA method according to the manufacturer’s guidelines (Elabscience Biotechnology Inc., USA). Testicular xanthine oxidase (XO) activities were based on a previously established method (22, 29).

### 2.11 Apoptotic markers

A spectrophotometric assay using diphenylamine (DPA) methods (14, 22) was employed in estimating the DNA fragmentation index, while testicular caspase 3 activities were estimated according to the manufacturer’s instructions (Elabscience Biotechnology Co., Ltd., USA).

### 2.12 Statistical analysis

Graph Pad Prism, version 7.00, was used for statistical analysis. To analyze data from various groups, one-way analysis of variance (ANOVA) was employed, followed by Tukey’s *post hoc* test for multiple comparisons. Data are presented as the mean ± standard error of the mean (SEM). P < 0.05 was considered statistically significant.

## 3 Results

### 3.1 Epididymal sperm parameters

O3FA ameliorated the BPF-induced decrease in sperm count, motility, and normal morphology compared with the control (**Table 1**). While there was no significant difference in abnormal sperm morphology and motility of rats treated with low and high doses of O3FA, animals treated with high doses of O3FA showed an improved sperm count compared with their counterparts treated with a low dose.

**Table 1:** Effect of BPF on sperm parameters.

Parameters	Control	BPF	O3FA-L	O3FA-H
Sperm Count (x106/ml)	9.760±0.143	6.300±0.148^a^	9.080±0.097^a,b^	9.640±0.093^b,c^
Motility (%)	86.00±0.548	63.40±0.510^a^	83.40±1.939^b^	86.00±1.095^b^
Abnormal Sperm Morphology (%)	79.578±1.047	49.895±1.894^a^	64.895±1.904^a,b^	68.904±1.894^a,b^

^a^p ^<^0.05 versus control, ^b^p < 0.05 versus BPF, ^c^p < 0.05 versus BPF + O3FA-L using one-way analysis of variance (ANOVA) followed by Tukey’s post hoc test for pairwise comparison. BPF, Bisphenol F; O3FA-L, omega-3 fatty acid low dose; O3FA-H, omega-3 fatty acid high dose.

### 3.2 Hormonal imbalance

As shown in **Table 2**, O3FA blunted the observed BPF-induced hormonal imbalance by significantly increasing serum LH (p<0.0001), FSH (p<0.0001), and testosterone (p<0.0001) and decreasing serum estradiol (p<0.0001) in BPF-exposed rats. Although low and high doses of O3FA significantly blunted the was observed BPF-induced hormonal imbalance, a more ameliorative effect was observed in animals treated with a high dose than their counterparts treated with a low dose.

**Table 2:** Effect of BPF on reproductive hormones.

Parameters	Control	BPF	O3FA-L	O3FA-H
Serum LH (mIU/mL)	6.125±0.129	2.500±0.321 ^a^	4.313±0.449 a^,b^	5.806±0.374^a,b,c^
Serum FSH (mIU/mL)	4.123±0.0648	2.677±0.149 ^a^	3.457±0.236 a^,b^	4.046±0.179 ^b,c^
Serum Testosterone (ng/mL)	2.29±0.083	1.229±0.068 ^a^	2.078±0.069 a^,b^	2.150±0.100 a^,b,c^
Serum Estradiol (pg/mL)	4.541±0.130	6.998±0.155 ^a^	4.889±0.138 a^,b^	4.956±0.149 a^,b^

^a^p ^<^0.05 versus control, ^b^p < 0.05 versus BPF, ^c^p < 0.05 versus BPF + O3FA-L using one-way analysis of variance (ANOVA) followed by Tukey’s post hoc test for pairwise comparison. BPF, Bisphenol F; O3FA-L, omega-3 fatty acid low dose; O3FA-H, omega-3 fatty acid high dose; LH, Luteinizing hormone; FSH, Follicle stimulating hormone.

### 3.3 Histopathological findings

**Figure 1:**
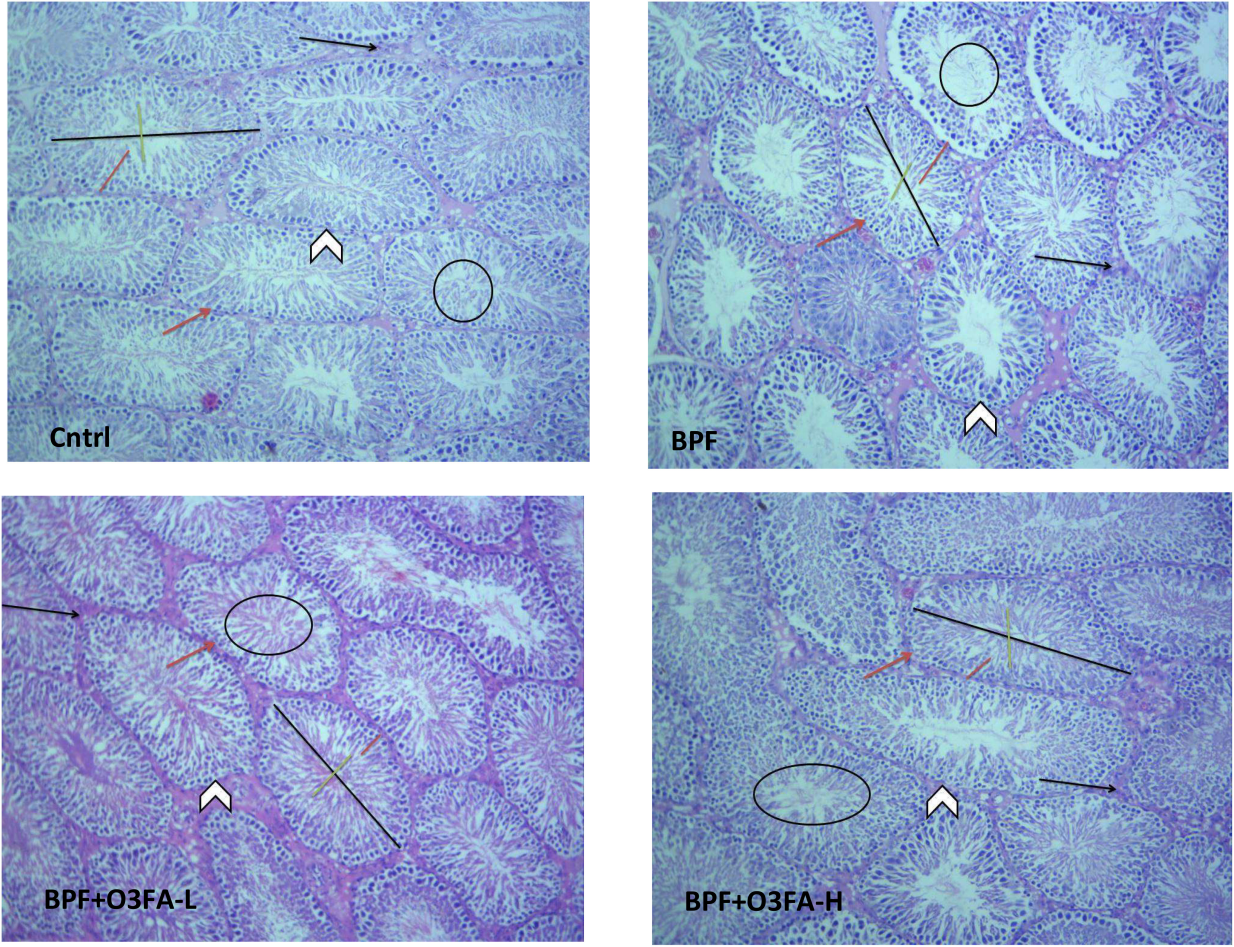
Cntrl: The testicular histoarchitecture appears preserved. The seminiferous tubules are normal with germ cells at varying degree of maturation (arrow head). The lumen of the seminiferous tubules shows normal sperm cells (black circle). The Sertoli cells appear normal (red arrow). The interstitial space appears normal with normal Leydig cell mass (black arrow). BPF: The testicular histoarchitecture appears distorted. The seminiferous tubules show germ cells at varying degree of maturation (arrow head). The lumen of the seminiferous tubules shows scanty sperm cells (black circle). The Sertoli cells appear reduced (red arrow). The interstitial space appears normal with reduced leydig cell mass (black arrow). BPF+O3FA-L and BPF+O3FA-H: The testicular histoarchitecture appears preserved. The seminiferous tubules are normal with germ cells at varying degree of maturation (arrow head). The lumen of the seminiferous tubules shows normal sperm cells (black circle). The Sertoli cells appear normal (red arrow). The interstitial space appears normal with normal leydig cell mass (black arrow). Black span: diameter of the seminiferous tubules; red span: epithelial height; green span: diameter of the seminiferous lumen. Stain H and E; x100. BPF, Bisphenol F; O3FA-L, omega-3 fatty acid low dose; O3FA-H, omega-3 fatty acid high dose.

As shown in **Figure 1**, BPF distorted the normal testicular histology, evidenced by a distorted histoarchitecture, scanty sperm cells in the lumen of the seminiferous tubule, and reduced Sertoli cells and Leydig cell mass compared with the control. This was accompanied by an increase in testicular histoarchitecture and seminiferous luminal diameter and a decrease in biopsy score, epithelial height, and seminiferous tubular diameter (**Table 3**). These observed alterations were ameliorated by co-administration of BPF with both doses of O3FA.

**Table 3:** Effect of BPF on testicular cytoarchitecture.

Parameters	Control	BPF	O3FA-L	O3FA-H
Testicular histoachitecture	1.333±0.211	3.500±0.224^a^	1.833±0.307^a,b^	1.500±0.224^b,c^
Testicular biopsy score	9.667±0.211	6.833±0.307 ^a^	8.667±0.211^a,b^	9.333±0.211^b,c^
Epithelial Height (µm)	68.910±3.863	40.020±2.065 ^a^	71.070±6.016^a,b^	73.910±2.050^a,b,c^
Seminiferous Tubular Diameter (µm)	324.7±10.39	189.3±7.35 ^a^	300.3±6.30^a,b^	336.0±19.54^a,b,c^
Seminiferous Luminal Diameter (µm)	38.26±5.102	141.5±5.440 ^a^	41.62±0.558^a,b^	43.36±3.518^a,b,c^

^a^p ^<^0.05 versus control, ^b^p < 0.05 versus BPF, ^c^p < 0.05 versus BPF + O3FA-L using one-way analysis of variance (ANOVA) followed by Tukey’s post hoc test for pairwise comparison. BPF, Bisphenol F; O3FA-L, omega-3 fatty acid low dose; O3FA-H, omega-3 fatty acid high dose.

### 3.4 Testicular injury markers

**Figure 2:**
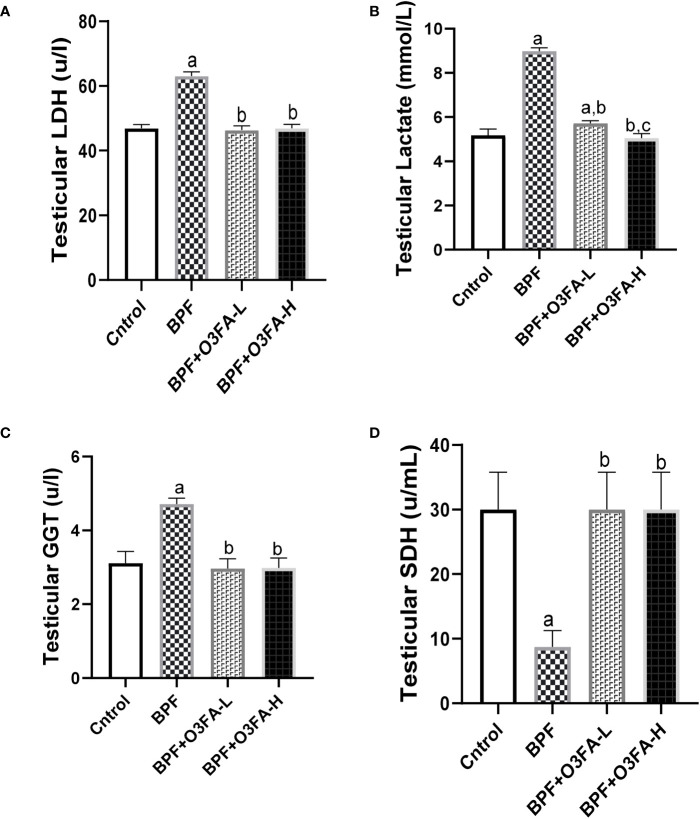
Effect of O3FA on testicular **(A)** LDH **(B)** Lactate **(C)** GGT **(D)** SDH in BPF exposed rats. ^a^p ^<^0.05 versus control, ^b^p < 0.05 versus BPF, ^c^p < 0.05 versus BPF + O3FA-L using one-way analysis of variance (ANOVA) followed by Tukey’s *post hoc* test for pairwise comparison. BPF: Bisphenol F, O3FA-L: omega-3 fatty acid low dose, O3FA-H, omega-3 fatty acid high dose; GGT, Gamma glutamyl transpeptidase; SDH, Sorbitol Dehydrogenase.

BPF exposure led to a significant increase in testicular LDH (p<0.0001), GGT (p<0.001), and lactate (p<0.0001) and a decrease in testicular SDH (**Figure 2**) compared with the control. In contrast, co-administration of BPF with low and high doses of O3FA prevented the observed alterations in testicular injury markers activities.

### 3.5 Steroidogenic enzymes

**Figure 3:**
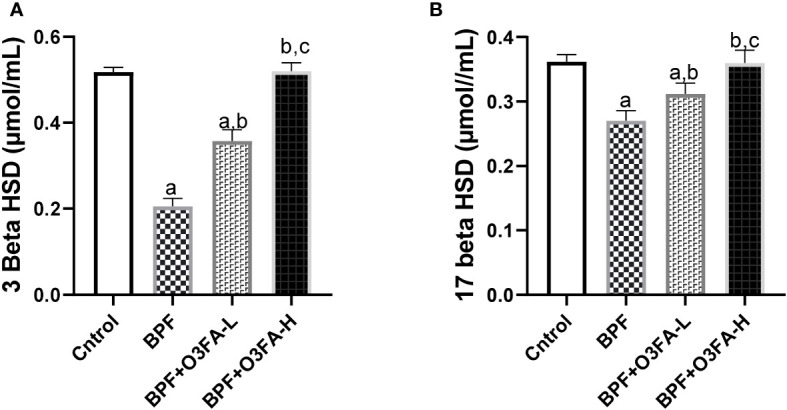
Effect of O3FA on testicular **(A)** 3 β-HSD **(B)** 17 β-HSD in BPF exposed rats. ^a^p ^<^0.05 versus control, ^b^p < 0.05 versus BPF, ^c^p < 0.05 versus BPF + O3FA-L using one-way analysis of variance (ANOVA) followed by Tukey’s *post hoc* test for pairwise comparison. BPF, Bisphenol F; O3FA-L, omega-3 fatty acid low dose; O3FA-H, omega-3 fatty acid high dose; 3 Beta HSD, 3-Beta–hydroxysteroid dehydrogenase; 17 Beta HSD, 17-Beta hydroxysteroid dehydrogenase.

BPF administration led to a significant decrease in 3β-HSD (p<0.001) and 17β-HSD (p<0.001) compared with the animals in the control group (**Figure 3**). This observed decrease was ameliorated by the co-administration of BPF with both doses of O3FA. Although both doses of O3FA blunted the observed decrease, the animals treated with high doses exhibited better ameliorative effects than their counterpart treated with low doses.

### 3.6 Inflammatory markers

**Figure 4:**
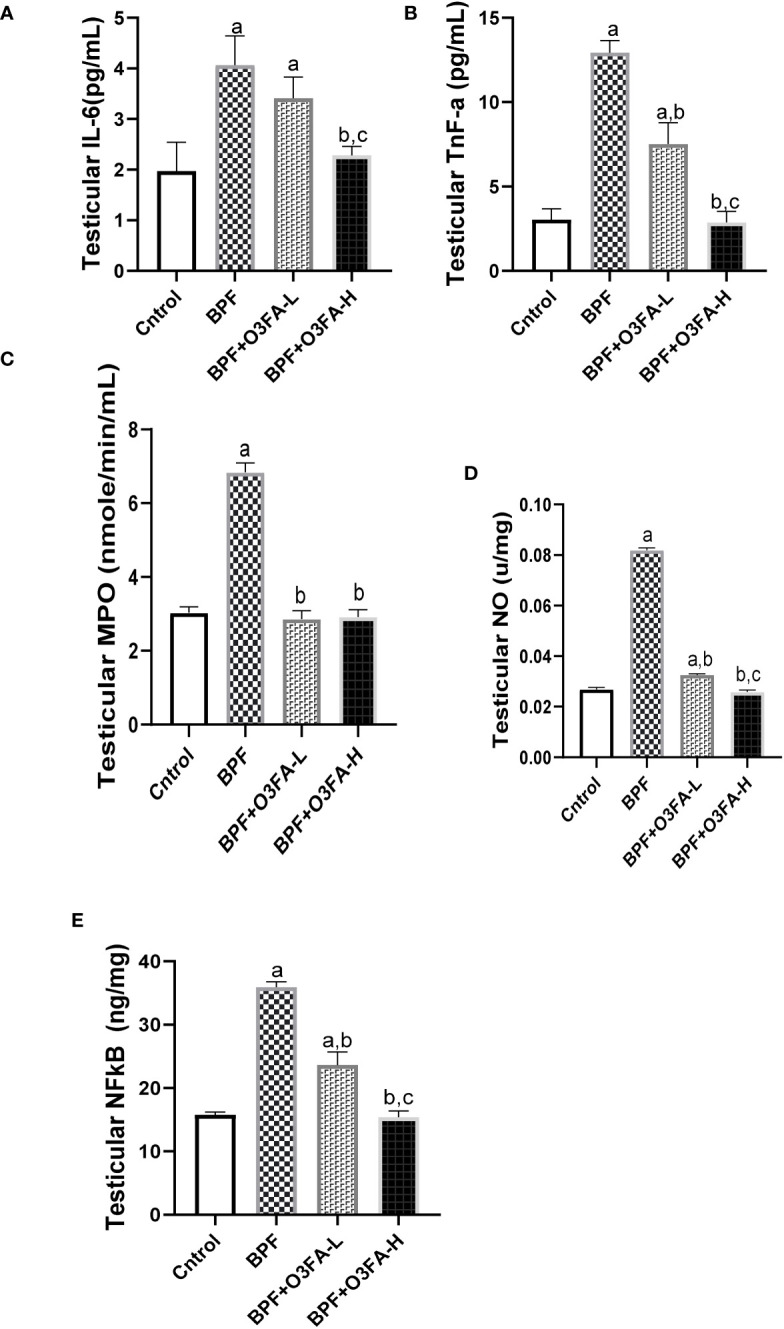
Effect of O3FA on testicular **(A)** IL-6 **(B)** TnF-α **(C)** MPO **(D)** NO **(E)** NFκB in BPF exposed rats. ^a^p ^<^0.05 versus control, ^b^p < 0.05 versus BPF, ^c^p < 0.05 versus BPF + O3FA-L using one-way analysis of variance (ANOVA) followed by Tukey’s *post hoc* test for pairwise comparison. BPF, Bisphenol F; O3FA-L, omega-3 fatty acid low dose; O3FA-H, omega-3 fatty acid high dose; IL-6, Interleukin-6; TnF-α, Tumor necrosis factor alpha; MPO, Myeloperoxidase; NO, Nitric Oxide; NFκB, Nuclear factor kappa-light-chain-enhancer of activated B cells.

Testicular IL-6 (p<0.0001), TnF-α (p<0.001), MPO (p<0.001), NO (p<0.001), NFκB (p<0.0001), and XO (p<0.001) were significantly increased in the animals treated with BPF alone compared with their counterparts in the control group (**Figure 4**). The observed increase was abolished by low and high-dose treatment of O3FA. The ameliorative effect of O3FA was more pronounced in animals treated with a high dose except in testicular MPO, where there was no significant difference between animals in the BPF+O3FA-L and BPF+O3FA-H group.

### 3.7 Oxidative stress markers

**Figure 5:**
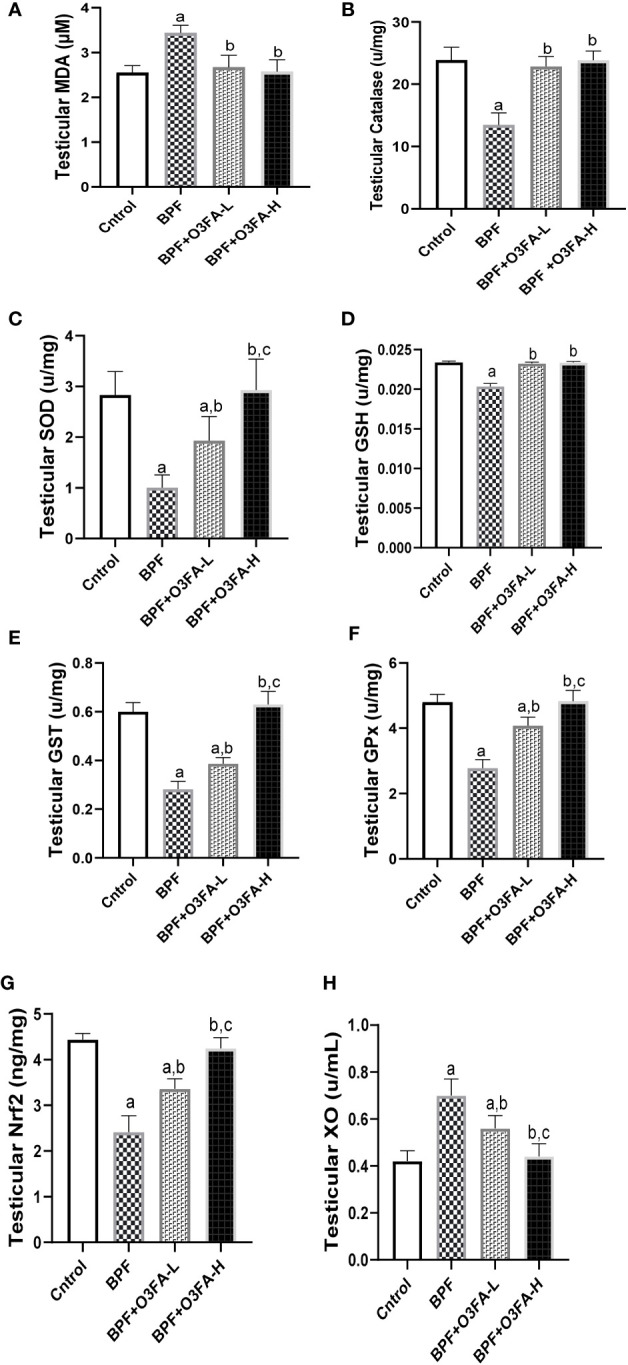
Effect of O3FA on testicular **(A)** MDA **(B)** Catalase **(C)** SOD **(D)** GSH **(E)** GST **(F)** GPx **(G)** Nrf2 **(H)** XO in BPF exposed rats. ^a^p ^<^0.05 versus control, ^b^p < 0.05 versus BPF, ^c^p < 0.05 versus BPF + O3FA-L using one-way analysis of variance (ANOVA) followed by Tukey’s *post hoc* test for pairwise comparison. BPF, Bisphenol F; O3FA-L, omega-3 fatty acid low dose; O3FA-H, omega-3 fatty acid high dose; MDA, Malondialdehyde; SOD, Superoxide dismutase; GSH, Glutathione; GST, Glutathione S-transferases; GPx, Glutathione peroxidase; Nrf2, nuclear factor erythroid 2–related factor 2, XO: Xanthine oxidase.

As shown in **Figure 5**, BPF exposure led to a significant increase in testicular MDA and a decrease in CAT (p<0.0001), SOD (p<0.0001), GSH (p<0.001), GST (p<0.001), GPx (p<0.001), and Nrf2 (p<0.0001) compared with the control. This observed increase in testicular pro-oxidant and decrease in testicular antioxidants was blunted by co-administration of BPF with low and high doses of O3FA.

### 3.8 Apoptotic markers

**Figure 6:**
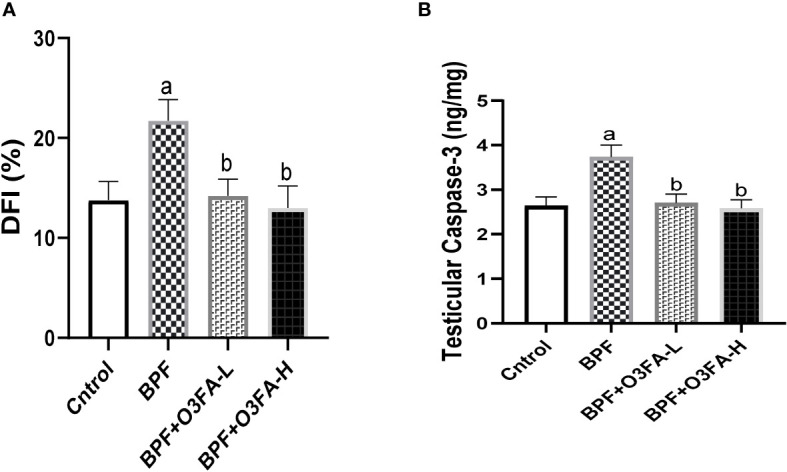
Effect of O3FA on testicular **(A)** DFI **(B)** Caspase 3 in BPF exposed rats. ^a^p ^<^0.05 versus control, ^b^p < 0.05 versus BPF versus BPF + O3FA-L using one-way analysis of variance (ANOVA) followed by Tukey’s *post hoc* test for pairwise comparison. BPF, Bisphenol F; O3FA-L, omega-3 fatty acid low dose; O3FA-H, omega-3 fatty acid high dose; DFI, DNA Fragmentation Index.

Bisphenol F administration significantly increased DFI (p<0.001) and caspase 3 (p<0.0001) activities compared with the control group (**Figure 6**). These observed increases in apoptotic markers were ameliorated by the co-administration of BPF and low and high doses of O3FA.

## 4 Discussion

This study showed that BPF exposure disrupted testicular functions and induced testicular injury in male Wistar rats. BPF-induced hormonal imbalance and impaired sperm quality were associated with impairment in steroidogenic enzyme activities, inflammation, and oxidative stress. The alterations were associated with impaired testicular cytoarchitecture and increased testicular injury markers activities. It was also accompanied by the Nrf2/NFκB pathway distortion and upregulation of caspase 3-mediated apoptosis. Also, this study established the protective role of O3FA in BPF-impaired sperm quality, hormonal imbalance, and oxido-inflammatory injury via the modulation of the Nrf2/NFκB pathway and repression of the caspase 3 pathway.

In the present study, a significant decline in sperm count, motility, and normal morphology of rats exposed to BPF was observed. Furthermore, there was a significant decrease in serum testosterone which was accompanied by a decline in steroidogenic enzyme activities, which are consistent with our previous findings (5, 7). Different mechanisms may explain the reduced sperm quality and circulatory testosterone. The impaired sperm parameters and decline in serum testosterone could be associated with the direct effect of BPF on the testicular tissue leading to male reproductive dysfunction (30). The findings from this study showed that BPF distorted the normal testicular histology by disrupting testicular histoarchitecture and reducing sperm cells in the lumen of the seminiferous tubule, which was accompanied by a distortion in testicular histoarchitecture, mean testicular biopsy score, seminiferous tubular and luminal diameter, and epithelial height. These suggest that BPF-impaired sperm quality via direct testicular damage. Also, BPF-impaired sperm quality and declined testosterone could be due to its endocrine-disrupting activities. The findings from this study that BPF disrupted the hypothalamic-pituitary-gonadal (HPG) axis are consistent with the findings of 31. The HPG axis forms a closed loop, and it is the major signaling pathway controlling reproductive hormone secretion (32). The hypothalamus produces gonadotropin-releasing hormone (GnRH), which stimulates the production of LH and FSH from the pituitary gland. LH is responsible for stimulating the synthesis of testosterone (steroidogenesis), and FSH stimulates sperm production (spermatogenesis) from the testis suggesting that the observed decline in sperm quality, serum testosterone, and steroidogenic enzymes activities could be via the endocrine disrupting activities of BPF.

Also, the findings that BPF impaired testicular functions via direct testicular cell damage are consistent with the observed increase in testicular injury markers. Testicular activities of LDH, GGT, and SDH are markers of energy balance, spermatogenesis, and Sertoli functions (16). The observed significant increase in testicular lactate following BPF exposure indicates energy imbalance (33) and could result from a BPF-induced increase in the activities of LDH, which is an index of testicular degeneration.

Redox balance plays an integral role in testicular functions (34), and a disturbance in the redox balance leads to oxidative stress. Oxidative stress can activate various transcription factors leading to the activation of inflammatory pathways (35–37). Antioxidant defense systems have been identified to protect against oxidative stress, and Nrf2 is the major transcription factor responsible for regulating redox balance (38). Nrf2 maintained redox balance by regulating antioxidant enzymatic activities responsible for detoxifying and eliminating ROS. In addition to its antioxidant activities, Nrf2 is an anti-inflammatory agent by inhibiting NF-κB activities. NF-κB is responsible for proinflammatory gene induction, which increases inflammatory response (39). The observed decrease in Nrf2 following BPF exposure in this study agrees with the findings of Zhou et al. (40), which associated BPF exposure with decreased Nrf2 expression. This may account for the observed increase in oxidative stress (evidenced by an increase in testicular MDA and a decrease in CAT, SOD, GSH, GST, GPx) and Nf-κB-mediated inflammatory response (evidenced by an increase in testicular IL-6, TnF-α, MPO, NO, and XO).

Furthermore, excessive ROS and inflammation collaborate to stimulate caspase 3-mediated apoptosis (41), which is a contributing factor to male infertility (42). Caspase-3 is a major player in apoptosis initiation because of its involvement in receptor-mediated and the mitochondrial pathway, which are the major apoptotic signal transduction pathways (43). The increase in testicular caspase 3 could explain the increase in testicular DFI in this study since both have been positively related (43). The observed increase in caspase-3 and DFI in this study is similar to the findings of Ferreira et al. (44), which reported an increase in apoptotic markers activities following BPF exposure. Hence, BPF impaired hormonal balance and sperm quality by inducing oxidative stress, inflammation, and apoptosis via the modulation of Nrf2/NF-κB signaling and caspase-3 mediated apoptosis.

Another important finding from this study is the beneficial role of O3FA in BPF-induced testicular dysfunction. The present study revealed that O3FA alleviated BPF-induced testicular damage by suppressing testicular injury markers, oxidative stress, inflammatory response, and apoptotic markers, thus improving sperm qualities, reproductive hormones synthesis, and testicular cytoarchitecture. Although this study demonstrates for the first time that O3FA ameliorates BPF-induced testicular dysfunction, these findings concurred with previous findings that reported the antioxidant (45), anti-inflammatory (46), and antiapoptotic (47) activities of O3FA. The observed redox balance and decreased levels of NF-κB, IL-6, Tnf-α, MPO, and XO in the testicular tissues of O3FA-treated animals possibly explained O3FA-driven repression of apoptotic markers via the upregulation of Nrf2 activities. The gonado-protective effect of O3FA was accompanied by the restoration of testicular histoarchitecture and function by preventing distortion of histoarchitecture, scanty sperm cells in the lumen of the seminiferous tubule, and reduced Leydig cell mass, and normalization of sperm qualities and reproductive hormones.

## 5 Conclusion

The results from this study showed that O3FA co-treatment suppressed hormonal imbalance, poor sperm quality, oxidative stress, inflammation, and apoptosis via the modulation of Nrf2/NF-κB signaling and caspase-3 mediated apoptosis in BPF-treated rats. These findings suggest a possible insight into the protective molecular mechanisms of O3FA against BPF-induced testicular dysfunction.

## 6 Limitations and future perspectives

The BPF-treated rats testicular histology showed reduced Sertoli and Leydig cell count, which could result from BPF-induced apoptosis, and the TUNEL assay would establish which of the cells were more affected. However, this special staining was not done. This limitation opens a grey area for future exploration. Nevertheless, the in-depth testicular planimetry analysis and quantitative Sertoli and Leydig cells count in this study strengthened our findings on the distortive activities of BPF on testicular histology and cells.

## Data availability statement

The original contributions presented in the study are included in the article/**Supplementary Material**. Further inquiries can be directed to the corresponding author.

## Ethics statement

The designed experimental protocol was approved by the University of Ilorin Review and Ethical Committee, and in accordance with the National Institute of Health guidelines using the guide for the care and handling of laboratory animals.

## Author contributions

AO: Conceptualization, Data curation, Formal Analysis, Funding acquisition, Investigation, Methodology, Project administration, Resources, Software, Supervision, Validation, Visualization, Writing – original draft, Writing – review & editing. LO: Conceptualization, Formal Analysis, Investigation, Methodology, Project administration, Supervision, Validation, Visualization, Writing – review & editing. WA: Formal Analysis, Investigation, Methodology, Project administration, Software, Validation, Visualization, Writing – review & editing.
