# Supplementary figures and images for: Omega-3 fatty acid ameliorates bisphenol F-induced testicular toxicity by modulating Nrf2/NFkB pathway and apoptotic signaling
Source: Front Endocrinol (Lausanne). 2023 Sep 20;14:1256154. doi: 10.3389/fendo.2023.1256154 (PMC10548221; doi:10.3389/fendo.2023.1256154)

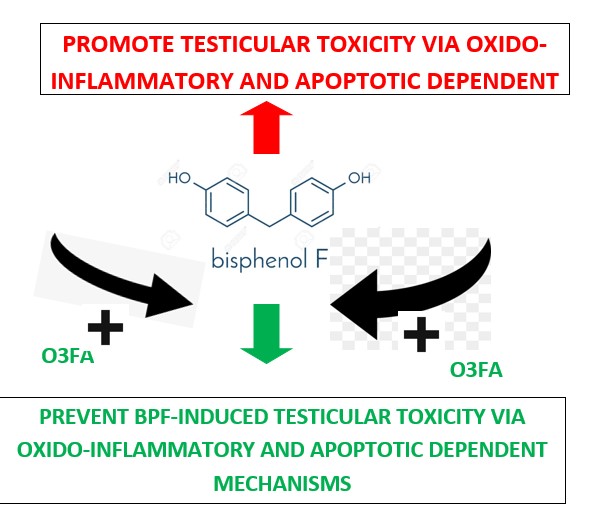

Supplement: Supplementary file 1 [file Image_1.jpeg]
